# Maximizing Onboard Hydrogen Storage Capacity by Exploring High-Strength Novel Materials Using a Mathematical Approach

**DOI:** 10.3390/ma17174288

**Published:** 2024-08-30

**Authors:** Andrei Ratoi, Corneliu Munteanu, Dan Eliezer

**Affiliations:** 1 Mechanical Engineering, Mechatronics and Robotics Department, Mechanical Engineering Faculty, “Gheorghe Asachi” Technical University of Iasi, 700050 Iasi, Romania; andrey_ratoi@yahoo.com (A.R.); cornelmun@gmail.com (C.M.); 2 Technical Sciences Academy of Romania, 26 Dacia Blvd., 030167 Bucharest, Romania; 3 Department of Material Engineering, Ben Gurion University of the Negev, 1 David Ben Gurion Blvd., Beer Sheva 8410501, Israel

**Keywords:** hydrogen storage, capillary arrays, high-strength materials, glass, gravimetric capacity, volumetric capacity

## Abstract

Hydrogen fuel holds promise for clean energy solutions, particularly in onboard applications such as fuel cell vehicles. However, the development of efficient hydrogen storage systems remains a critical challenge. This study addresses this challenge by exploring the potential of high-strength novel materials, including glass, to maximize onboard hydrogen storage capacity. A mathematical approach was employed to evaluate the feasibility and efficacy of various high-strength materials for hydrogen storage. This study focused on capillary arrays as a promising storage medium and utilized mathematical modeling techniques to estimate the storage capacity enhancement achievable with different materials. The analysis revealed significant variations in storage capacity enhancements in different high-strength novel materials, with glass having promising results. Glass-based materials demonstrated the potential to meet or exceed US Department of Energy (DOE) targets for both gravimetric and volumetric hydrogen storage capacities in capillary arrays. By leveraging a mathematical approach, this study identified high-strength novel materials, including glass and polymers, capable of substantially improving onboard hydrogen storage capacity: 29 wt.% with 40 g/L for quartz glass and 25 wt.% with 38 g/L for Kevlar compared to 5.2 wt.% with 26.3 g/L from a conventional type IV tank. These findings underscore the importance of material selection in optimizing hydrogen storage systems and provide valuable insights for the design and development of next-generation hydrogen storage technologies for onboard applications.

## 1. Introduction

Hydrogen, a well-known chemical element, holds significant promise as a green energy source for a sustainable future [1]. Its versatility is truly remarkable, allowing for storage in various forms such as its physical state (liquid or gas) or material-based (stored on the surface of or within solids), which can be tailored to meet the specific requirements of diverse applications [2]. However, one of the most pressing challenges is the development of compact, robust, and safe storage solutions capable of accommodating a wide range of portable applications. This is crucial to the field of transportation, where the demand for clean and efficient energy sources is steadily growing [3].

Compact and reliable storage systems are essential to ensuring the safety of passengers and the environment. They are also necessary for maximizing the driving range and overall performance of hydrogen vehicles. These storage systems must achieve a delicate balance between their capacity, safety, and practicability. This is so as to meet the unique demands of various transportation applications including scooters, cars, buses, trucks, trains, ships, and airplanes.

As the automotive industry increasingly turns its focus toward hydrogen fuel cell technology [4], there is a growing urgency to develop storage solutions that can store hydrogen with high energy density while also being lightweight and durable. Such innovations are mandatory to expand the adoption of hydrogen-powered vehicles and reduce their reliance on fossil fuels.

**Figure 1:**
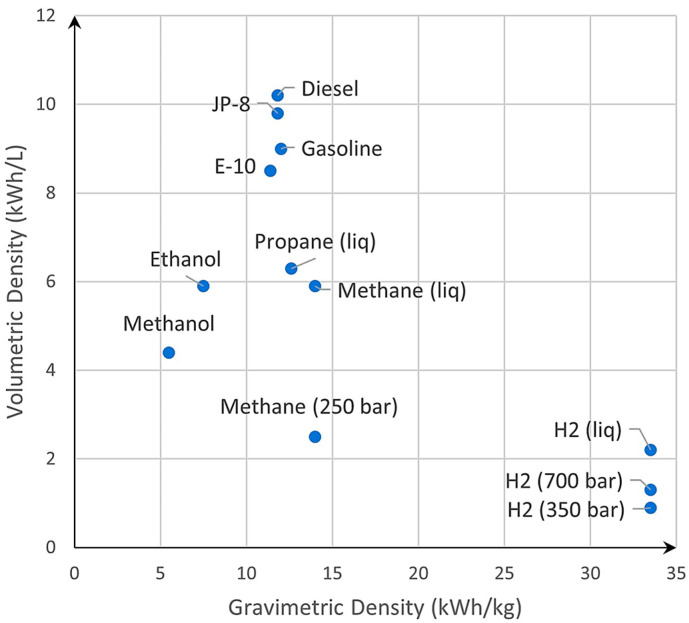
Comparison of volumetric and gravimetric energy densities in different fuel types [5].

**Figure 2:**
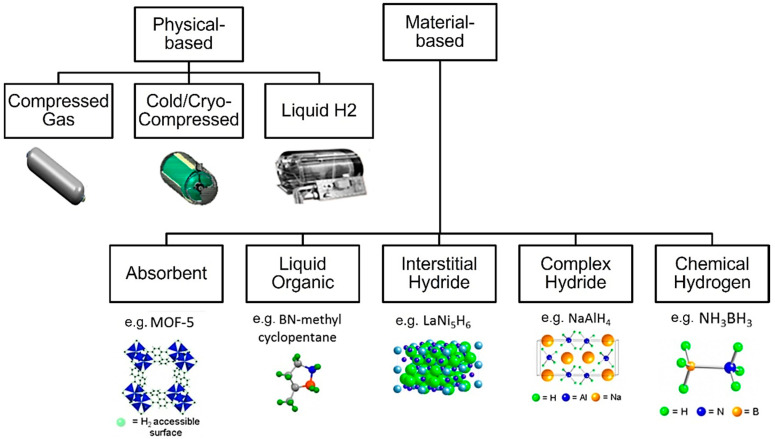
Overview of existing technical solutions for storing hydrogen [8].

Compared to conventional fuels such as diesel or gasoline, hydrogen offers a significant advantage in gravimetric density, providing nearly three times the energy for the same mass (120 MJ/kg for H_2_ compared to around 40 MJ/kg for diesel/gasoline). In practical terms, this means that 1 kg of hydrogen can yield the same amount of energy as approximately 3 kg of diesel or gasoline (as illustrated in Figure 1). However, the challenge arises when it comes to volumetric capacity. Storing the equivalent amount of energy with hydrogen demands nearly five times the volume in comparison to conventional fuels (around 35 MJ/L for diesel/gasoline compared to 1–2 MJ/L for H_2_). For example, the energy contained in just 1 L of diesel would require approximately 4.2 L of storage space when using liquid hydrogen and even more for compressed gas (as depicted in Figure 2). This being said, the importance of maximizing hydrogen storage capacity for onboard applications cannot be overstated. Efficient storage systems are pivotal for extending the range and performance of fuel cell vehicles, thereby accelerating their transition toward sustainable transportation solutions.

Despite significant progress in the field, several challenges and controversies persist. Diverging hypotheses about the optimal material properties and storage mechanisms underscore the complexity of the hydrogen storage problem. Moreover, achieving the ambitious targets set forth by the US Department of Energy (DOE) [6] for gravimetric and volumetric hydrogen storage capacities remains a formidable challenge, requiring innovative approaches and novel materials.

When tackling these challenges, reducing the volume occupied by hydrogen is imperative. Achieving this reduction involves subjecting hydrogen to higher compression pressures, which demands the utilization of specialized high-strength materials [7]. These materials must do more than exhibit remarkable strength; they should also possess a low density to ensure that their storage weight remains at a minimum. The rigorous assessment of materials based on their unique combination of strength and low density is the key point of the proposed systematic approach. Through this method, we endeavor to pinpoint materials that hold the promise of revolutionizing hydrogen storage, making it more efficient and practical for a wide array of applications.

This study aims to address the critical need for enhanced hydrogen storage solutions by exploring the potential of high-strength novel materials within the context of capillary arrays. Through a comprehensive mathematical analysis, we assessed the feasibility and efficacy of various materials in maximizing onboard hydrogen storage capacity, obtaining higher gravimetric and volumetric capacities than standard type I–IV tanks and showing high potential to exceed the ultimate DOE targets for hydrogen storage capacity. Our findings provide valuable insights into material selection strategies and offer promising avenues for the advancement in hydrogen storage technology in onboard applications.

## 2. Materials and Methods

### 2.1. Analysis of Existing Hydrogen Storage Solutions

This study started with a comprehensive examination and analysis of existing technical solutions for hydrogen storage, comprising a broad spectrum of approaches utilized in both academic research and industrial applications.

Based on the existing literature [9,10,11,12,13,14,15,16,17,18,19,20,21,22,23], hydrogen can be stored in various forms such as material-based states using hydrides and metal–organic frameworks (MOFs), in liquid form using vessels, or in gas in pressure vessels. In Figure 2, a classification of hydrogen storage methods is presented. The solutions are divided into two main categories:

*Physical-based Storage*

1. Compressed Gas—storing hydrogen at high pressures in specially designed tanks. The image shows a cylindrical tank, likely made of reinforced materials to withstand high pressure [16].
2. Cold/Cryo-Compressed—combines compression with cooling. The image shows a more complex tank with insulation, likely to maintain the low temperatures required [17].
3. Liquid Hydrogen—hydrogen is stored in liquid form at extremely low temperatures (around −253°. The tank shown is larger and more elaborate, reflecting the need for significant insulation and temperature control [18].

*Material-based Storage*

1. Absorbent—exemplified by MOF-5, showing a crystalline structure with nodes and linkers creating a porous framework where hydrogen molecules can be absorbed [19].
2. Liquid Organic—exemplified by BN-methyl cyclopentane, whose molecular structure is made of carbon, nitrogen, and boron, forming a cyclic compound that can reversibly store hydrogen [20].
3. Interstitial Hydride—illustrated by LaNi_5_H_6_, a structure where hydrogen is integrated within the interstices of a metal alloy composed of lanthanum and nickel [21].
4. Complex Hydride—represented by NaAlH_4_, a complex crystal structure with sodium, aluminum, and hydrogen atoms arranged in a specific pattern [22].
5. Chemical Hydrogen—exemplified by NH_3_BH_3_, forming a molecular structure with nitrogen, boron, and hydrogen atoms bonded together [23].

Each storage technology has advantages and disadvantages. While physical methods offer simplicity, they may require high pressures or very low temperatures. The material-based methods can potentially store more hydrogen by volume but may face challenges in hydrogen release. Ultimately, the key focus should be on their suitability for portable devices in fuel cell applications or internal combustion engine applications as the primary energy carrier.

One of the most challenging parameters in hydrogen storage today is achieving high gravimetric and volumetric capacities. To address this, the US Department of Energy (DOE) defined specific targets to be achieved by 2020, 2025, and an ultimate target [6].

**Figure 3:**
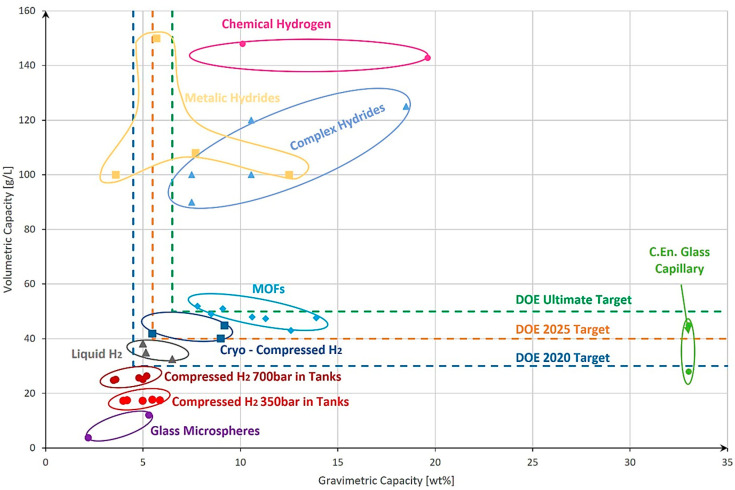
Comparison between different methods of storing hydrogen and the corresponding gravimetric and volumetric capacities.

To support the analysis of the existing hydrogen storage systems, an overview was created (Figure 3). For this, the following information was collected from the literature and datasheets [9,10,11,12,13,14,15,16,17,18,19,20,21,22,23]: a technical solution, the gravimetric capacity, and the volumetric capacity. The next step was to cluster the identified solutions based on the state, material-based, liquid, and gas, and represent them in a graph as points using the gravimetric and volumetric capacities as coordinates for the x- and y-axes. Elliptical shapes were introduced in the graph to group different values obtained from different sources and should not be considered immutable. Additionally, Figure 3 provides the target values set by the DOE [6].

Based on this analysis, conventional storage systems, such as compressed hydrogen in tanks, fall below the DOE’s 2020 targets. Liquid hydrogen solutions achieve the 2020 target, while cryo-compressed hydrogen has already reached the proposed target for 2025. Solid-state hydrogen storage technologies appear to be reaching and even exceeding the DOE’s ultimate target.

However, it is important to note that liquid hydrogen and cryo-compressed hydrogen face a significant challenge because of their extremely low working temperatures [24], which have a substantial economic impact when considering large-scale and affordable applications. On the other hand, material-based storage solutions (MOFs, hydrides), despite offering better gravimetric storage capacities, are hindered by poor absorption and desorption kinetics, as well as their requirement of high temperatures for their release process [24]. Compressed hydrogen in tanks (from type I to type IV) [25] at 350 bar or 700 bar remains the most well-known and practical technology. Although it presents lower storage capacities when compared to other technologies, it shows promise as an active research field through the development of innovative approaches to hydrogen storage. As shown in Figure 3, glass capillary arrays developed by C.En [26,27] already achieved the DOE’s 2025 target and have the potential to reach the ultimate target through the optimization of their material properties and array geometry. Based on the observations above, the solution to storing hydrogen in glass capillaries will be considered in our study.

In compressed gas hydrogen storage, a principal drawback is the large volume of tanks needed. To reduce this volume, higher compression pressure is necessary, which leads to the need for special materials with high strength. Additionally, to maintain a low storage weight, the chosen material should possess a low density. Consequently, the next step in optimizing gas hydrogen storage is the identification of novel materials that offer the best combination of density and strength, thereby addressing the challenges associated with compactness, robustness, and safety in hydrogen storage systems.

### 2.2. Analysis of Existing Material Types

After selecting the hydrogen storage technology with high potential to be used at a larger scale for onboard applications, the next step was to identify a novel material that provided the optimal mechanical properties to support a safe and compact storage solution.

**Figure 4:**
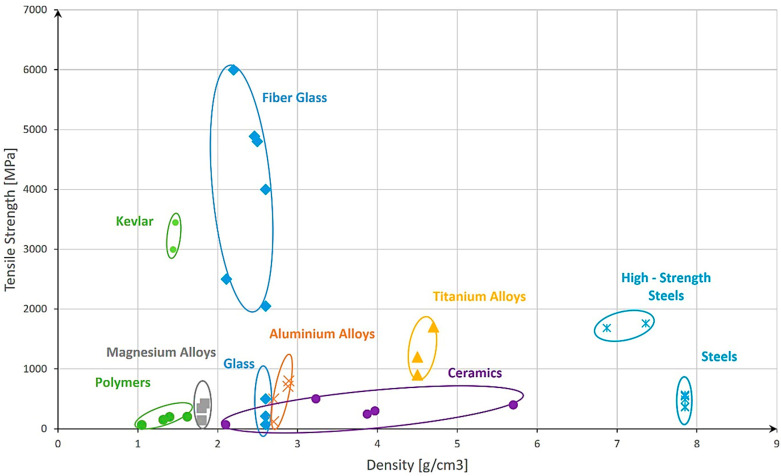
Comparison between the density and tensile strength of different types of materials.

For a proper evaluation of the most suitable material, an overview comprising different types of materials, their density, and tensile strength was created (Figure 4). The first step was to identify the main groups of materials that could be used, including steels, ceramics, glass, polymers, and alloys. Afterward, based on the literature and material datasheets, a collection of technical details like the material type, density, and tensile strength [28,29,30,31,32,33,34,35,36,37] was composed. Next, the materials were placed in the graph using the density and tensile strength for the x- and the y-axes and afterward clustered with elliptical shapes. The ellipses were only used for better visualization of the material groups and should not be considered immutable.

Upon analysis, it was evident that steels exhibit relatively good strength and are commonly used materials. However, their high-density results in a significant increase in their storage weight, which can be undesirable for practical applications. Furthermore, steels [38] are susceptible to embrittlement in the presence of hydrogen gas, which poses sustainability concerns for certain applications.

On the other hand, polymers offer a remarkable advantage in terms of density, with values approximately 7–8 times lower than those of steels. However, their strength is relatively low. An interesting solution arises with the use of Kevlar, which exhibits nearly twice the tensile strength of the strongest steel. That said, Kevlar presents challenges in terms of hydrogen permeation resistance [39] over time and being an expensive material. Moreover, its processing properties may pose difficulties in certain manufacturing processes.

A surprising and promising finding emerges when considering glass as a potential material for gas hydrogen storage systems. Glass exhibits approximately four times lower density than steel, and when reduced to fiber size, its strength surpasses one of the strongest steels. The strength of glass fibers can reach up to two times that of Kevlar. Additionally, promising capabilities in terms of hydrogen permeation are present [40,41]. These exceptional properties position glass as a potential novel material for gas hydrogen storage systems.

### 2.3. Analysis of Existing Glass Types

**Figure 5:**
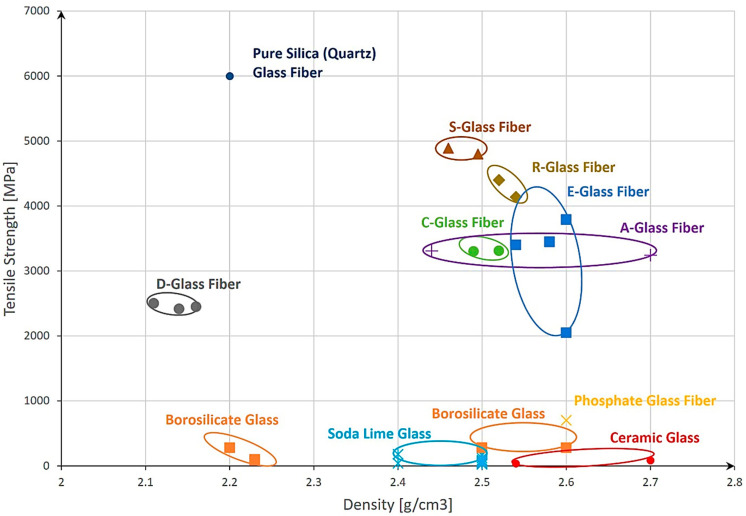
Comparison between the density and tensile strength of different types of glasses [43].

After the analysis and selection of the most promising material type, in our case glass, the next step was to determine the most suitable glass type. To accomplish this, the Interglad Glass Database [42] was consulted to identify various types of glass with different compositions. Similar to the previous analysis, the tensile strength and density of these glass types were evaluated, and based on the datasheets, the glass parameters are represented in Figure 5. The grouping of them was completed using elliptical shapes for better visualization and should not be considered immutable.

Based on Figure 5, a significant improvement in tensile strength was observed when transitioning from bulk/plate-shaped glass to fiber-shaped glass. This suggests that by reducing the vessel to a capillary form, the resulting tensile strength should exhibit remarkable improvements when compared to standard vessels commonly used for gas storage. In terms of density, it is worth noting that borosilicate glass demonstrated the lowest values. Borosilicate glass has been widely utilized in various applications due to its advantageous properties, such as its high thermal resistance [44] and low coefficient of thermal expansion [44]. Its low density further supports its potential suitability as a material for gas hydrogen storage systems.

Due to its impressive mechanical properties, quartz glass has been considered to be able to highlight the maximum storage capacities that can be obtained with this potential material.

### 2.4. Storage Capacity Evaluation

**Figure 6:**
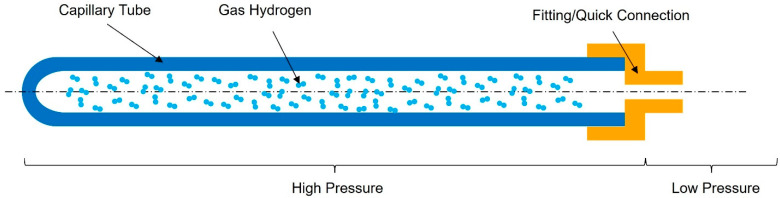
Capillary tube hydrogen storage concept (longitudinal section cut).

In the next phase of this research, the aim was to assess the potential gravimetric and volumetric capacities of a hydrogen storage system. To achieve this, a hydrogen storage configuration comprising a single cylindrical capillary tube was considered (Figure 6).

**Figure 7:**
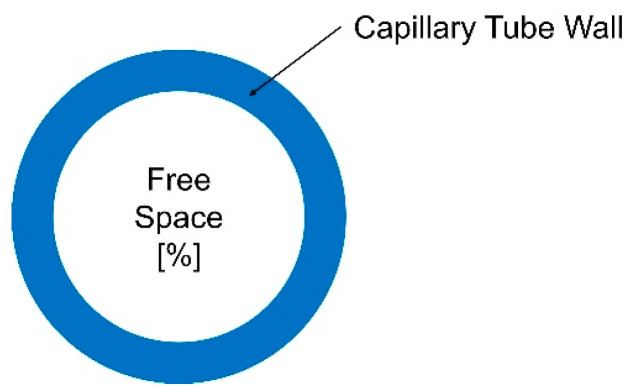
Capillary tube (transverse section cut).

To facilitate the evaluation of the relationship between the inner diameter, wall thickness, and outer diameter, the concept of “free space” is introduced. Free space refers to the percentage of a tube section that can be filled in with hydrogen relative to the total section of the tube (Figure 7).

Free space can be calculated using the following equation:

(1) $$\mathrm{FS}=\frac{{R_{i}}^{2}}{{R_{e}}^{2}}\times 100\left[\%\right]$$

where R_e_ represents the external radius of the capillary and R_i_ is the internal radius of the capillary.

**Figure 8:**
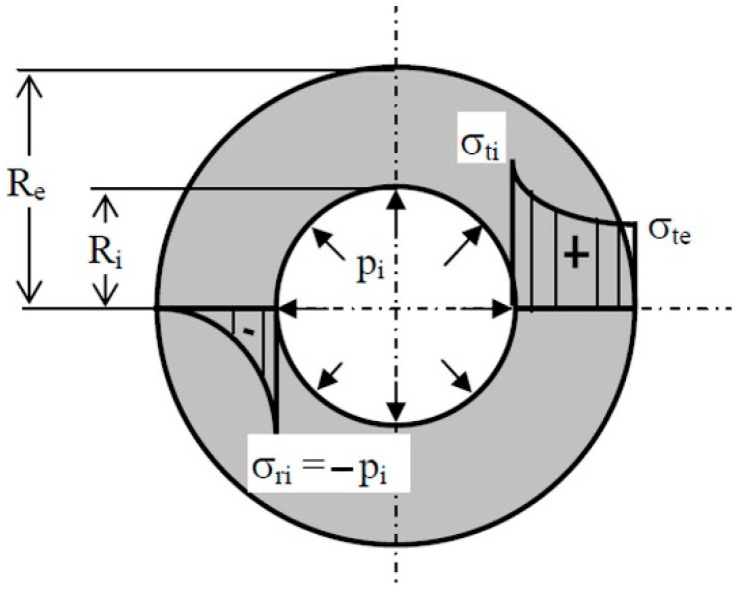
Tensile strength variation in the vessel wall [45].

Based on the figure below (Figure 8), the stress on the vessel wall when the inner pressure is applied can be calculated with the equation below [45]:

(2) $$\sigma_{\mathrm{tmax}}=\frac{\left({R_{i}}^{2}+{R_{e}}^{2}\right)\cdot p_{i}}{{R_{e}}^{2}-{R_{e}}^{2}}\left[\mathrm{MPa}\right]$$

Other important parameters that needed to be defined were the gravimetric and volumetric capacities.

1. Gravimetric capacity—represents the amount of hydrogen that can be stored per unit mass of storage. It is a measure of the efficiency of hydrogen storage in terms of weight. Higher gravimetric capacity indicates a greater amount of hydrogen that can be stored while minimizing the weight of the storage system and can be calculated using the following equation:

(3) $$G_{\mathrm{capacity}}=\frac{m_{H2}}{m_{H2}+m_{\mathrm{storage}}}\times 100\;\left[\mathrm{wt}.\%\right]$$

where

- m_H2_—the mass of hydrogen calculated by multiplying the density of hydrogen at 700 bar and the volume of hydrogen inside the capillary tube (V_H2_ = πR_i_^2^L, where L is the length of the capillary tube)
- m_storage_—the mass of storage calculated by multiplying the density of storage material and the volume of storage (for a capillary tube V_storage_ = V_capillary_ = πR_e_^2^L, where L is the length of the capillary tube)

2. Volumetric capacity—represents the amount of hydrogen that can be stored per unit volume of storage. It reflects the ability to store a larger quantity of hydrogen within a given spatial volume. Higher volumetric capacity indicates a more compact storage system capable of storing greater amounts of hydrogen. The parameter can be calculated using the following equation:

(4) $$V_{\mathrm{capacity}}=\frac{m_{H2}}{V_{\mathrm{storage}}}\;\left[\frac{g}{L}\right]$$

To facilitate a comprehensive comparison of materials and identify novel options for optimizing hydrogen storage capacities, a set of input data and preconditions were used. These considerations served as the basis for evaluating and selecting the most suitable materials for hydrogen storage. The considered factors were the following:

- 700 bar inner pressure load for the capillary tube;
- safety factor of 2;
- theoretical tensile strength of the capillary tube—considered only in the tangential direction—without longitudinal stress when having closed-ended cylinders.

For our investigation, the following materials were selected: from Figure 3, steel was chosen for comparison, due to its common usage in pressure vessels. The steel type with the highest ultimate tensile strength (UTS) was selected to ensure the maximum strength of the capillary tube possible. The same approach was used for polymers. Here, Kevlar was selected due to its highest UTS from this material category. The third material was quartz glass selected from Figure 4 based on its highest UTS.

## 3. Results

### 3.1. Free Space Influence on Capillary Tube Wall Stress

**Figure 9:**
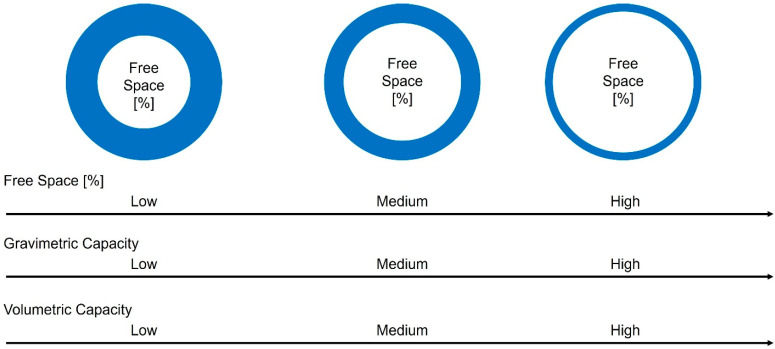
Influence of free space on the gravimetric and volumetric capacities.

The influence of variations in the free space parameter on the gravimetric and volumetric capacities is illustrated in Figure 9. This analysis provides valuable insights into optimizing the storage system for enhanced performance and efficiency.

By carefully adjusting the free space parameter, it was possible to achieve a balance between maximizing the amount of hydrogen stored and minimizing the overall volume occupied by the storage system. This is a crucial aspect in the development of efficient and practical hydrogen storage solutions.

According to the representation in Figure 9, as the free space parameter increases, the wall thickness of a capillary tube becomes thinner. This results in the creation of a larger space within the tube for hydrogen storage. Consequently, the gravimetric capacity is significantly enhanced, as a larger quantity of hydrogen can be stored within a capillary tube of reduced weight, facilitated by the thinner walls.

Moreover, the volumetric capacity of the storage system also experiences a notable increase. This is attributed to the larger available space for hydrogen within the capillary tube, while the outer dimensions of the tube remain unchanged. By optimizing the free space parameter, the storage system can achieve a higher volumetric capacity, effectively maximizing the amount of hydrogen that can be stored within a given volume.

These observations highlight the importance of carefully selecting and optimizing the free space parameter to achieve the desired balance between gravimetric and volumetric capacities. By finetuning this parameter, it becomes possible to enhance both the amount of hydrogen stored and the overall efficiency/practicality of the hydrogen storage system.

To evaluate the maximum allowable free space for different materials, an analysis was carried out by calculating the tensile stress exerted on the vessel wall using the provided Equation (2) and considering the permissible stress values for each material, as indicated in Table 1. This theoretical estimation insight into the potential free space, calculated with Equation (1), can be achieved while ensuring the structural integrity of the storage system.

By progressively increasing the free space parameter and keeping constant the inner pressure of hydrogen, different stress on the capillary wall was obtained. In our calculation, a single cylindrical capillary tube was considered. The results are summarized below.

**Figure 10:**
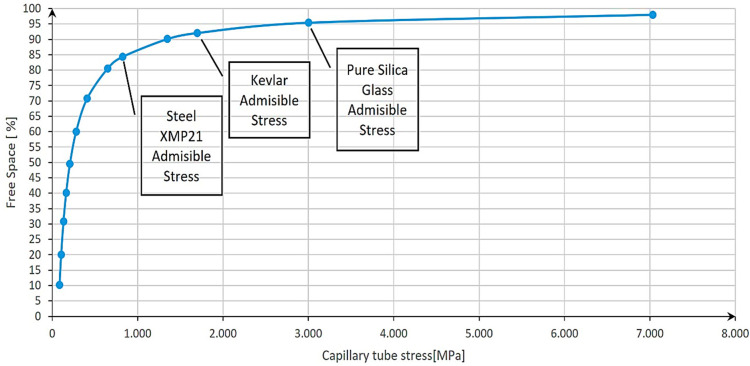
Capillary tube stress variation relative to free space at 700 bar of storage pressure.

According to Figure 10, and considering the admissible stress of the chosen materials from Table 1, the maximum free space that can be achieved by the analyzed materials is the following:

Based on the result from Figure 10, it was observed that the highest-strength steel could only achieve a maximum of 84% of free space to be considered safe at 700 bar storage pressure. Afterward, the curve tended to flatten and an increase with only 10% of free space would lead to the need for a material that resists at three times higher stress than steel. This impressive performance can be achieved only by glass.

Utilizing the maximum achievable free space for various materials from Table 2, the theoretical estimation of gravimetric and volumetric capacities could be calculated. This analysis provides valuable insights into the potential storage performance of different materials and aids in the identification of optimal choices for hydrogen storage systems.

### 3.2. Gravimetric Capacity

The evaluation of the gravimetric capacity was performed by progressively increasing the free space using Equation (1) (keeping the outer diameter of the capillary tube constant and increasing the inner diameter) and considering the calculation of the gravimetric capacity using Equation (3). The graph below was created as a function f(G_capacity_) = FS. The limitation of each material related to the free space was taken from Table 2 and the influence of the material on the gravimetric capacity was conducted by the different weights of the storage in Equation (3). In our calculation, a single cylindrical capillary tube was considered. The dashed lines in the graph represent the DOE targets [6].

**Figure 11:**
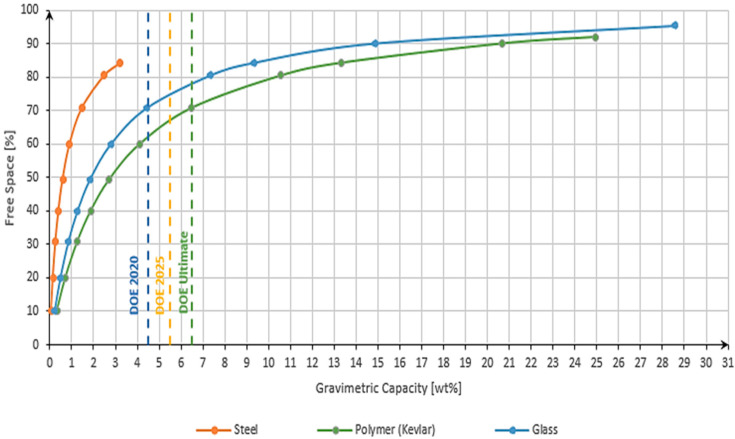
Influence of free space relative to gravimetric capacity at 700 bar of storage pressure.

The analysis of Figure 11 revealed important insights regarding the achievable gravimetric capacity of different materials. When utilizing one of the highest-strength steels available, it became evident that the maximum gravimetric capacity achievable was approximately 3 wt.%. This limitation arose primarily from the high density of the material, which restricted the amount of hydrogen that could be stored per unit weight. Consequently, even the target set for 2020 by the Department of Energy (DOE) cannot be reached with steel as the storage material.

On the other hand, the results for the highest-strength polymers and glass demonstrated remarkable performance. These materials exhibited gravimetric capacities that surpassed the DOE’s ultimate target by a factor of 4–5. This exceptional achievement underscores the immense potential of polymers and glass as viable options for hydrogen storage in the future. Their lower densities allow for a higher volume of hydrogen to be stored while maintaining a reasonable weight, resulting in significantly improved gravimetric capacities.

Based on the results obtained, with polymer, a maximum of 25 wt.% could be achieved, and with glass, almost 29 wt.%.

These findings highlight the significance of exploring alternative materials, such as high-strength polymers and glass, to enhance the gravimetric capacity of hydrogen storage systems. By surpassing the DOE’s ultimate target, these materials offer promising opportunities for advancing the efficiency and performance of hydrogen storage technologies.

### 3.3. Volumetric Capacity

For the volumetric capacity evaluation, a similar method was performed. By progressively increasing the free space using Equation (1) (keeping the outer diameter of the capillary tube constant and increasing the inner diameter), the volumetric capacity was calculated using Equation (4) as a function of f(V_capacity_) = FS. The influence of different materials is linked to the fact that higher free spaces lead to a higher amount of hydrogen contained in a capillary.

**Figure 12:**
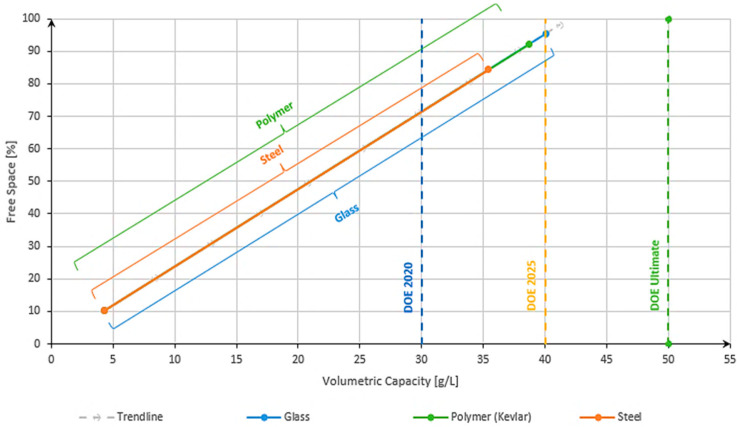
Influence of free space relative to volumetric capacity at 700 bar of storage pressure.

Figure 12 highlights that in terms of volumetric capacity, steel exhibited the capability to surpass the DOE 2020 target. This is a good achievement, but not enough for today’s needs. However, when considering the DOE 2025 target, it became apparent that none of the materials examined, except for glass, can meet the requirement. Glass stands out as a potential solution due to its ability to accommodate higher free spaces, thereby enabling a greater storage capacity for hydrogen within the same volume. Additionally, polymers should not be neglected as they are also close to the DOE 2025 targets. Based on the results obtained, with polymer material used, a maximum of 38 g/L of volumetric capacity was obtained, and for a glass capillary, around 40 g/L.

**Figure 13:**
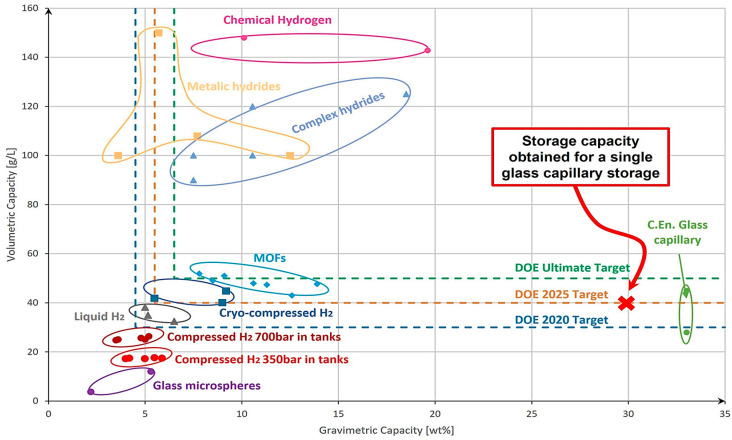
Storage capacity obtained for a single glass capillary compared to other storage technologies.

The mapping of the result obtained based on the analysis compared to other existing storage solutions can be observed in Figure 13.

## 4. Discussion

The basic analysis used in this study addressed whether there could be a technical solution that could safely and practically achieve the DOE [5] target in terms of gravimetric and volumetric capacities opening the door to a new era of clean transportation. The presented results clearly showed that the performance of standard hydrogen compressed tanks at 700 bar could be exceeded by using glass capillary storage systems. While standard type I–IV tanks can achieve a maximum of 5 wt.% and around 30 g/L, the glass capillary solution leads to 29 wt.% and 40 g/L storage capacities, which represent an improvement of +500% in terms of gravimetric capacity and +30% in terms of volumetric capacity. This is mainly related to the extraordinary strength of glass when reduced to being fiber-sized compared to all other materials and combined to an almost four times lower density compared to steels, leading to an improved weight of storage made of glass. Parallel to these outcomes, a promising technical solution arose from polymer capillary tubes. Polymer-based systems present, in certain ranges of free space, better results in terms of their gravimetric capacity than glass due to the lower density of polymer, but the ultimate limit is exhibited by glass due to its higher ultimate strength allowing higher free space.

In essence, both glass and polymer exhibit impressive strength-to-weight ratios and can be tailored to specific hydrogen storage requirements. It is important to note that these evaluations were based on a singular capillary tube configuration. While glass shows promise in its volumetric capacity, further exploration is warranted to optimize its performance and identify novel solutions. Capillary arrays, for instance, could offer enhanced volumetric capacity by leveraging interconnected networks of capillaries to maximize the available storage volume within a given space. Investigating the potential of capillary arrays holds promise for achieving the DOE ultimate target and further advancing the volumetric capacity of hydrogen storage systems.

## 5. Conclusions

Glass capillary hydrogen storage has emerged as a potential game changer in the field of energy, showing results that could meet the Department of Energy (DOE) targets and surpass the performance of standard-type I–IV tanks in terms of both gravimetric and volumetric capacities. Theoretical calculations suggested that a storage system utilizing a single glass capillary tube could achieve remarkable metrics: a gravimetric capacity of 29 wt.% and a volumetric capacity of 40 g/L. This represented a significant leap forward, with approximately 500% higher gravimetric capacity and 30% higher volumetric capacity compared to conventional type I–IV tanks.

While glass shows the most promise, high-strength polymers also demonstrate impressive potential. A single capillary storage system made from the highest-strength polymer could theoretically achieve a maximum gravimetric capacity of 25 wt.% and a volumetric capacity of 38 g/L. In contrast, even the highest-grade steel falls short in comparison, with a single capillary storage system made of steel reaching a maximum of only 3.2 wt.% gravimetric capacity and 35 g/L volumetric capacity.

These findings position capillary tube-based hydrogen storage as one of the most promising technical solutions for achieving the highest gravimetric capacity among all current technical solutions. However, pushing the boundaries of performance requires careful material selection and design optimization. For instance, increasing the free space (the volume available for hydrogen storage) from 85% to 95% necessitates a material with triple the strength. Currently, only glass and high-strength polymers can support free space percentages higher than 85%.

The research points toward the need for further investigation into capillary arrays. This approach could potentially enhance storage capacity by maximizing free space while simultaneously reducing the overall weight of a storage system. Such advancements could mark a significant step forward in the development of efficient, high-capacity hydrogen storage solutions, crucial for the broader adoption of hydrogen as a clean energy carrier.

## Figures and Tables

**Table 1:** Overview of the materials and properties considered for the calculations.

Material	Density [g/cm^3^]	UTS [MPa]	σ Admissible [MPa] *
Steel XMP 21	6.87	1683	842
Pure silica quartz fiber	2.2	6000	3000
Polymer (Kevlar 149)	1.47	3450	1725

* The admissible stress was calculated by dividing the UTS of the material by the safety factor 2.

**Table 2:** Maximum free space allowable for the selected materials.

Material	Free Space [%]
Steel XMP 21	84
Polymer (Kevlar 149)	92
Pure silica quartz fiber	95

## Data Availability

The calculated data from this study can be provided by the corresponding author upon request.
